# Habit of elongate amphibole particles as a predictor of mesothelial carcinogenicity

**DOI:** 10.1016/j.toxrep.2025.101908

**Published:** 2025-01-14

**Authors:** Andrey A. Korchevskiy, Ann G. Wylie

**Affiliations:** a Chemistry & Industrial Hygiene, Inc., 7333 W. Jefferson Ave., Suite 235, Lakewood, CO 80235, USA; b University of Maryland, Geology Building, 8000 Regents Drive. #237, College Park, MD 20742, USA

**Keywords:** Mesothelioma, Amphiboles, Dimensional Coefficient of Carcinogenicity (DCC), Asbestiform, Membranolytic toxicity index (HC50)

## Abstract

**Introduction:**

Amphiboles are a class of minerals that are abundantly present in the environment. Amphiboles may exist in several habits, with asbestiform particles behaving like typical amphibole asbestos and non-asbestiform (or massive) reported to be less biologically active.

**Materials and methods:**

The available dimensional information for 16 testing sets (8 asbestiform and 8 non-asbestiform types of tremolite) was combined. In addition, three validation sets (an asbestiform sample from Eastern New York and non-asbestiform samples from Quebec and Falls Village, Connecticut) were tested by Transmission Electron Microscopy (TEM) to determine dimensional distribution. Mathematical modeling was utilized to determine the classification method for amphiboles with various habits.

**Results:**

The decision boundary method was developed to distinguish asbestiform vs. non-asbestiform samples (with error rate of 0 % for single-sourced tremolite and 3 % for potentially mixed samples). All validation datasets were correctly classified. A new empirical dimensional coefficient of carcinogenicity (DCC) was proposed, with DCC = 1 - exp(-0.11 Surface Area /(1000width^3^ + 1)). For several mineral types (crocidolite, amosite, Libby amphiboles, anthophyllite, chrysotile, and erionite), it was demonstrated that mesothelioma potency factors can be predicted based on DCC and biosolubility with a high level of accuracy (R=0.98, R^2^=0.96, p < 0.006). It was demonstrated that modeled mesothelioma potency correlates with relative potency for pleural instillation in Wistar rats, and correlates inversely with membranolytic toxicity index HC50. Mesothelioma potency was demonstrated to be negligible in all non-asbestiform sets.

**Conclusions:**

The habit of amphibole particles is predictive of biological behavior that can be estimated from the dimensional data for the particles.

## Highlights

• The decision boundary method was developed to distinguish asbestiform vs. non-asbestiform samples.
• The coefficient 1 – exp(-0.11 *x* Surface Area /(1000width^3^ +1)) predicts mesothelial toxicity.
• Modeled mesothelioma potency correlates with relative potency for pleural instillation in rats.
• Mesothelioma potency was demonstrated to be negligible in all non-asbestiform sets.

## Graphical Abstract

**Figure:**
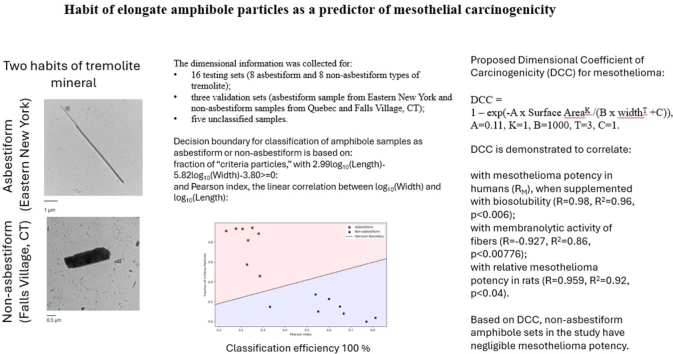

## 1 Introduction

Amphiboles are a group of chain-silicates widely spread in nature, with possible toxic effects closely related to the habit of the minerals [1]. It is typical for amphibole occurrences to be represented as asbestiform and non-asbestiform based on differences in their morphology, specific crystallographic properties, and dimensions of the particles in soil, rock, or airborne samples [2], [3].

Application of mechanical forces to non-asbestiform bulk amphibole material causes formation of so called “cleavage fragments”: elongate non-asbestiform amphibole particles that may partly overlap in dimensions with some asbestos fibers, but do not possess the same morphological and other physical properties as asbestos. Also, major dimensional characteristics of cleavage fragments result in quite different biological reactions when non-asbestiform particles and asbestiform fibers are introduced to cells, tissues, and organisms [4], [5]. In some settings, cleavage fragments can be mixed with true asbestos fibers. The characteristic example is Libby amphibole, which contaminated the vermiculite mine in Libby, Montana (MT) with tremolite being one of the components of the solid solution series of amphibole occurring there, and which also includes the amphiboles winchite and richterite. At Libby, the particles can be both asbestiform and non-asbestiform, though fibrous. Brown and Gunter [6] reported that 36 % of Libby amphibole fibers were asbestiform (or “asbestos”) by morphology vs. about 33 % fragments and 31 % “not classified”.

Verkouteren and Wylie [7] provided detailed analysis for systematic relationship among cell parameters, composition, optical properties, habit, and evidence of discontinuity for 103 samples from the tremolite-actinolite-ferro-actinolite series. About 33 % of datasets were classified as asbestiform, 43 % as massive (non-asbestiform), and 24 % belonged to a socalled “byssolitic” habit referring to a variety of brittle, glassy amphiboles that are fibrous but not asbestiform,

Many authors reported that asbestiform and non-asbestiform amphiboles have different biological potential. Nolan et al. [8] demonstrated that epidemiological, in vivo and in vitro tests are evidence of the significant differences between tremolite with various habits. Gamble and Gibbs [9] determined that a cohort of workers exposed to non-asbestiform tremolite and anthophyllite had no excess risk of lung cancer or mesothelioma, in contrast to elevated risk level observed in cohorts exposed to commercial asbestos.

In this regard, it is important to analyze further the differences between non-asbestiform and asbestiform varieties of amphibole in order to establish the characteristics that make their carcinogenicity potential different. Not only can this analysis bring more insight into the mechanisms of carcinogenicity for elongate mineral particles, but it is also expected to allow for development of a methodology to distinguish between asbestiform and non-asbestiform, and therefore between carcinogenic and non-carcinogenic particles. It is especially relevant for mesothelioma carcinogenicity, as that remains a public health priority. Mesothelioma is a cancer that develops in the mesothelial tissue of the pleura, peritoneum, or testis. Elongate particles (specifically, asbestos and asbestiform varieties) play an important role in the origination and development of mesothelial tumors, though a fraction of idiopathic or spontaneous cases is also significant (especially in women) (WHO/IARC, 2021). In contrast, spherical dust particles are almost never reported as able to produce these types of tumors in humans.

This paper will explore varieties of amphiboles from different sources to demonstrate differences between the mineralogically predetermined habit of elongate particles. We used tremolite mineral samples as one of the most representative examples of amphiboles, which occurs in a variety of habits [10], [11], [12], [2], [3], [13], [7]. In particular, Cavariani [14] noted that the non-asbestiform variety of tremolite is “predominant” in the earth’s crust, though asbestiform tremolite can also be found “almost everywhere” in the world. Tremolite is common in carbonate-rich rock and in and around mafic and ultramafic rock (rocks high in Mg and Fe) that have been metamorphosed during mountain building processes.

It should be noted, however, that asbestiform tremolite deposits have not had much commercial significance, primarily because they tend to be small in comparison to amosite, anthophyllite asbestos, and crocidolite; and reports of the mining and use of tremolite as a commodity in the last century are scarce. Its widespread occurrence and habit variety make tremolite a useful mineral to study, and in our mineral database we have many examples.

We will demonstrate that the habit of tremolite for a specific source can be efficiently predicted by the dimensional characteristics of elongate particles including distributions of length and width, and the relationship between these two parameters. We will use mathematical modeling to develop a method for prediction of mesothelioma potency for various habits and sources of tremolite. We will show that the habit of tremolite significantly affects various characteristics of particles that correspond to observed differences in biological behavior.

Based on the studies of tremolite, a new quantitative parameter of elongate particles will be proposed as a tool for classification of particles as related to their effect on mesothelioma development. Dimensional Coefficient of Carcinogenicity or DCC is a metric that correlates with mesothelioma potency of various mineral types of elongate mineral particles, and also discriminates between asbestiform and non-asbestiform habits of amphibole particles with acceptable statistical efficiency. Introduction of the new metric helps to demonstrate that habit of particles is an essential characteristic, defining their toxicological behavior. Using DCC, it is possible to evaluate exposure to elongate mineral particles from the position of their propensity to cause mesothelioma. It is especially important for public health and occupational safety applications, when naturally occurring asbestos (NOA) is found in airborne samples. It will demonstrate that mesothelioma risk should not be expected to be elevated when the habit of amphibole particles is non-asbestiform, and DCC is not exceeding a threshold value.

We used asbestiform and non-asbestiform tremolite samples that were analyzed and characterized previously to develop the classification models. Also, specifically for this study, we determined the dimensional distribution of several samples of tremolite from sources that were not well characterized previously. These samples were used for validation of the proposed models and their implications.

We utilized new modeling and statistical approaches to the analysis of toxicological characteristics for elongate mineral particles. In particular, we used Mahalanobis distance to evaluate relative proximity of datasets with length and width of particles as two variables. Mahalanobis distance has recently been described as one of the promising methods for biological and medical studies [16], [15]; however, we apparently utilized it first in the study of mesothelial cancers and asbestos.

In general, scientific literature in recent years has reached important advances in the area of asbestos toxicology. Nel [17] determined important systemic similarities between elongate particles and carbon nanotubes. Mossman [18] reviewed toxicity mechanisms of elongate mineral particles in the context of risk assessment. Roberts et al. [19] showed that cosmetic talc consumption was not associated with incidence of mesothelioma in the United States, confirming that talc itself and potential associated minerals in its deposit are not expected to be mesothelial carcinogens.

Our study is expected to outline a methodological basis for the differentiation of amphiboles by their habit as the important toxicological determinant. Habit of amphiboles is one of the keys to understanding mesothelial carcinogenicity which may be utilized in further toxicological and medical research, as well as in occupational health and environmental protection policies.

## 2 Materials and methods

Tremolite samples from the dimensional database described previously [5], [4] were selected. The database contains the largest and the most representative selection of elongate particles from various locations with dimensional characteristics. All tremolite samples from the database are included for this study. Habit of the datasets was determined by a professional mineralogist (Dr. Ann Wylie) based on the overall approach described in Verkouteren and Wylie [7] and Wylie et al. [5].

### 2.1 Testing datasets

The samples from specific tremolite occurrences with definite and mineralogically confirmed habit were used as testing datasets. Only samples from typical asbestiform and non-asbestiform habits were considered for inclusion in the testing group.

Testing datasets are listed in Table 1.

**Table 1:** Testing datasets for the study.

Source/location/sample	Habit
Udaipur, India	Asbestiform
Lone Pine, California (CA), USA	Asbestiform
Yamaga Mine, Japan	Asbestiform
Jamestown, CA, USA	Asbestiform
Korea	Asbestiform
HSE sample (1)	Asbestiform
HSE Sample (2)	Asbestiform
Metsovo, Greece	Asbestiform
Balmat, New York (NY), USA	Non-asbestiform
Miners Bay, Canada	Non-asbestiform
Gouverneur, New York, USA	Non-asbestiform
Sparta, New Jersey (NJ), USA	Non-asbestiform
Madagascar	Non-asbestiform
Shinness, Scotland	Non-asbestiform
Dornie	Non-asbestiform
Swansea	Non-asbestiform
NIOSH cleavage fragments	Non-asbestiform

From all testing datasets, 78.9 % of elongate mineral particles were measured by transmission electron microscopy (TEM), and 21.1 % by scanning electron microscopy (SEM) (assuming particles longer than 5 μm, with width (diameter) > =0.05 μm, and < =3 μm, with aspect ratio not less than 3:1). While SEM is known to overlook some of the narrowest fibers, especially in the case of chrysotile, we use population means and for that reason we believe the effect is small.

### 2.2 Validation datasets

Three datasets, not previously analyzed and/or described in literature, were selected for validation. Samples not analyzed previously were obtained from the collection of the Department of Geology at the University of Maryland. Habit was determined by mineralogical assessment as described earlier.

Sample vials were rinsed out into a specimen cup with 100 ml of deionized fiber free water. The container was capped and vigorously agitated for 10 seconds to break up any clumps of bulk material. The sample was then placed in a sonicator for 10 minutes, and a final vigorous agitation was applied for 10 seconds. The sample was allowed to settle for 2 minutes to allow agglomerations of consolidated material to fall to the bottom of the container. A 10 ml aliquot was then taken from the center of the sample and placed in a new unused sample container and brought up to 100 ml with deionized fiber free water. After homogenization, dilutions were made until a proper loading was achieved for TEM analysis.

The filter was dried and mounted onto glass microscope slides. Wedges of the filter were collapsed by hot acetone vapor and plasma etched in an oxygen plasma. A thin layer of carbon was deposited on the surface of the filter, thus imbedding the fibers of the sample in the carbon. The carbon-coated sample was then positioned onto a copper TEM grid and placed into an acetone Jaffe Wick where the filter membrane was dissolved. The sample replica, embedded in the carbon film and suspended on the grid, was placed in the TEM for analysis.

The samples were analyzed on JEOL 1011 analytical TEM equipped with a digital camera and Oxford Energy Dispersive X-Ray Analyzer (EDXA). A total of 200 fibers were recorded for each sample. The fiber counting rules and documentation requirements from ISO 10312:2019 method (Ambient air – Determination of Asbestos Fibres – Direct Transfer Transmission Electron Microscopy Method) were used to size and identify the fibers.

Validation datasets are listed in Table 2.

**Table 2:** Validation datasets for the study.

Tremolite source/location/sample	Habit
Eastern New York	Asbestiform
Falls Village, (CT), USA	Non-asbestiform
Quebec, Canada	Non-asbestiform

**Fig. 1:**
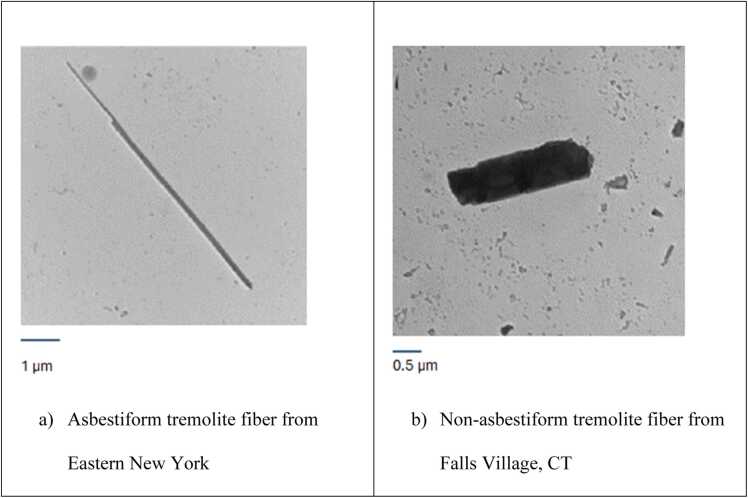
Microphotographs of tremolite fibers from validation sets.

The microphotographs of fibers from validation sets in TEM samples are demonstrated in Fig. 1.

It should be noted that no precise location for the tremolite sample from Quebec is known. The sample was obtained and analyzed earlier as a part of Verkouteren and Wylie study (2000), when it was determined as massive (non-asbestiform) by mineralogical characterization.

### 2.3 Unclassified datasets

Several samples were conditionally identified as “unclassified”. Tremolite from Brazil is usually considered asbestiform, but, as was discussed with Dr. Eric Chatfield (Chatfield 2024, personal communication), it appears to be “fibrous but not carcinogenic” and is probably the byssolitic type. The Ala di Stura, Italy sample was determined to be byssolitic by Verkouteren and Wylie [7]. The El Dorado Hills, California tremolite could contain both asbestiform and non-asbestiform material. Verkouteren and Wylie [7] evaluated the Barstow, California tremolite as asbestiform; however, the National Institute of Standards and Technology (NIST) in its certificate for standard reference material 1867 indicated that some fraction of the material was massive, probably evidence of different habits of tremolite from this location. Libby, Montana amphiboles were also included in the analysis for comparison purposes. Libby amphiboles mineralogically are not equivalent to tremolite, but contain some tremolite, and are most probably characteristically close enough to tremolite. Notably, prior to the current classification system for amphiboles, the solid solution there is reported as “soda tremolite”. See Verkouteren and Wylie [7] for a discussion of the nomenclature of “Libby amphibole”.

Unclassified datasets are listed in Table 3.

**Table 3:** Unclassified datasets for the study.

Sample source/location/sample	Habit
El Dorado Hills, CA	Unclassified
Ala di Stura, Italy	Unclassified
Brazil	Unclassified
Barstow, CA	Unclassified
Libby, MT amphiboles	Unclassified

### 2.4 Basic dimensional metrics used for the analysis

For each of the mineral occurrences from Table 1, Table 2, Table 3, statistical analysis was performed and the geometric mean length, width, and aspect ratio of EMPs in the populations were calculated, along with standard deviations. In general, length is the measurement of the longest dimension and width is the measurement of the longest dimension perpendicular to length of the two-dimensional particle observed under a microscope.

The Pearson index was used based on the regression equation

(1) Log W = F log L+C

where W is the width and L is the length of the particles. The fibrosity index for this equation (slope factor F) was described earlier [20], [21]. The Pearson index is a modification of the fibrosity index, allowing evaluation of the statistical independence of length and width of particles observed in the populations of asbestiform fibers, but not in mineral fragments. For the calculations of the Pearson index, we use fibers longer than 2 μm, with length/width> =3, width> =0.05 μm, and width< =3 μm.

Parameter EMPA was calculated as in Wylie et al. [22] who showed a close correlation between the abundance of particles of a specific size (Length>5 µm and width≤ 0.15 µm) and mesothelioma risk factors. EMPA is estimated as a fraction of EMPs with length/width> =3:1, length> 5 µm, and width< =0.15 µm, to all EMPs with length/width> =3:1 and length> 5 µm. For the calculations, we used particles with width> =0.05 μm and width< =3 μm.

The EMPA concept is aligned with earlier work by Stanton et al. [23] that selected a category of long, thin fibers as predictive of mesothelioma risk (Stanton’s hypothesis related mesothelioma mortality with fibers longer than 8 µm and thinner than 0.25 µm). Boron [24] suggested that fibers longer than 5 µm and thinner than 0.25 µm are responsible for mesothelioma toxicity. The toxicological significance of EMPA metric was confirmed by several publications during previous years [25], [26], [4].

Surface area of each of the particles was estimated for non-serpentine particles as

(2) Surface Area = 2LW + 2LTh+ 2WTh

where L = length, W = width, and Th = thickness,

and for serpentine as

(3) Surface Area = 0.5πW^2^ + πWL.

Thickness of particles (Th) was determined by the following empirical algorithm:

for erionite, Th=W,

for balangeroite, Th=W/1.1,

for fibrous talc Th=W/3,

for asbestiform and mixed amphiboles log10(Th)= 0.692log10(W)-0.493,

for other non-serpentine EMPs Th=W/1.9.

Aerodynamic diameter (AD) of each particle was calculated by the formula derived by Timbrell [27]:

(4) AD = 66 W (AR/(2+4 AR))^2.2^ x (ρρ/ρ_0_)^0.5^

where AD = aerodynamic diameter, W = measured width of the EMP, AR = length/width, ρρ = density in gm/cm^3^, and ρ0 = 1.0 gm/cm^3^. The density of minerals is provided as basic information in descriptive mineral texts. The influence of small density variations (second decimal place) on AD is minor, so the use of literature values for mineral compositions is reasonable even if the exact composition can only be approximated from a qualitative chemical analysis or precise determination of the indices of refraction.

### 2.5 Criteria particles

According to Wylie et al. [5], we determine “criteria particles” as those fitting the equation:

(5) 2.99log10(Length)-5.82log10(Width)-3.80> =0.

Criteria particles are those that this function associates with asbestiform habit. The fraction of criteria particles is determined for all EMPs with length> 5 μm, width> =0.05 μm, width< =3 μm, and length/width> =3:1. We define non-criteria particles as those that would not adhere to Eq. (5).

Eq. (5) shows the separation between asbestiform and non-asbestiform particles based on a “corrected” ratio of length and width. We can interpret Eq. (5) as a statement that Corrected Ratio =Length^2.99^/Width^5.82^ should be higher in asbestiform particles than in non-asbestiform variety. Earlier it was demonstrated that mesothelioma potency of various types of fibers correlates with the ratio Length^1.9^/Width^3^, and lung cancer potency with Length^0.4^/Width^1.2^ [26]. Eq. (5) for separation of asbestiform and non-asbestiform particles is close to the third power of the median of these potency-predictive ratios.

### 2.6 Mesothelioma potency factors for various types of minerals

Mesothelioma potency factors for pure tremolite are not reported in epidemiological studies. To estimate mesothelioma carcinogenicity of various samples of tremolite, we utilized characteristics of other minerals with available health-related information. Table 4 contains mesothelioma potency values R_M_ (%) determined by the Hodgson, Darnton method [28] that we used.

**Table 4:** Mesothelioma potency and characteristics of various mineral types of fibers.

Mineral type/ Locations	Mesothelioma potency, R_M_ (%)	Biosolubility, Years	Width, μm		Surface area, μm	
			Mean	Standard Deviation	Mean	Standard Deviation
Chrysotile (Quebec)	0.0014 *	0.3	0.37	0.0009	21.5	0.91
Amosite (South Africa)	0.11 *	74	0.475	0.0086	14.48	0.86
Crocidolite (Australia and South Africa)	0.52 *	66	0.31	0.0004	13.47	0.45
Anthophyllite (Russia)	0.056 * *	245	0.83	0.016	25.08	1.57
Libby amphiboles (Libby, MT)	0.03 *	49	0.66	0.009	21.97	0.91
Erionite (Karain, Turkey)	4.67 * **	181	0.45	0.01	24.94	1.16

*[28]

** [29]

*** [30]

### 2.7 Dimensional coefficient of carcinogenicity (DCC)

Empirical dimensional coefficient of carcinogenicity (DCC) is a new metric proposed to evaluate the mesothelioma carcinogenic potency of biodurable, rigid, elongate mineral particles based only on their dimensional characteristics.

DCC is proposed in the following general form:

(6) DCC = 1 – exp(-A x Surface Area^K^ /(B x width^T^ +C)).

Eq. (6) is empirical and is derived from the assumption that carcinogenic potential of elongate particles can be proportional to the ratio of surface area to some power of width. The exponent in the formula is used to put the value on probabilistic scale, changing from 0 to 1, depending on the potency.

The concept of DCC is based on the idea that mesothelioma carcinogenicity of biodurable and rigid elongate mineral particles can be characterized by their dimensions. Korchevskiy and Wylie [26] demonstrated that mesothelioma potency factors correlate with specific surface area (SSA), equal to the surface area of fibers divided by their volume. Specific surface area is closely approximated by reciprocal width; mathematically, SSA can be seen as a ratio of surface area and third power of width. The concept of DCC allows expansion of this relationship, proposing several coefficients to account for a more complex relationship between dimensional characteristics and potency. Biopersistence (reversed to biosolubility of particles) can be an additional factor determining the quantitative level of carcinogenicity. Also, elongate particles with low rigidity are not expected to produce mesothelioma, even if its DCC would be substantial. Therefore, DCC can be seen as one of the important, but not exclusive, characteristics of mesothelioma risk.

Based on that, we hypothesize that mesothelioma potency factors for various mineral occurrences can be approximated by DCC and the biosolubility of the minerals, measured in terms of the lifetime of fibers in a biological solution [31]. Values of the biosolubility of fibers are provided in Table 4, along with average width and surface area of particles longer than 5 μm, with width (diameter) > =0.05 μm and < =3 μm, and with aspect ratio not less than 3:1 as determined according to the dimensional database described in Wylie et al. [5]. Biosolubility for Libby amphiboles is reported as for tremolite.

In this case, mesothelioma potency can be seen as a function of DCC and biosolubility. In a simplified way,

(7) Log10(R_M_) = f(log10(Biosolubility), log10(DCC)),

where Biosolubility is used in terms of data from Table 4,

f – a function with two arguments, log10-transformed biosolubility and log10-transformed DCC.

We determined the numerical coefficients of Eq. 6 based on the following considerations.

It was assumed that DCC values should yield the highest linear correlation for Eq. 7 in the mineral occurrences from Table 4 (Condition 1). We also assumed that DCC should provide the greatest difference between asbestiform and non-asbestiform fibers in our study (Condition 2).

The following fitting coefficient (Coef) was used:

(8) Coef= R x U_asb_^1/4^ /U_non-asb_^1/2^,

where R – linear correlation coefficient for Eq. (7),

U_asb_ – average DCC value for asbestiform fibers for the testing datasets in our study,

U_non-asb_ - average DCC value for non-asbestiform particles for the testing datasets in our study.

For Conditions 1 and 2 to be fulfilled, the parameter Coef in Eq. (8) should be maximized. The powers in Eq. (8) are conditional, with linear correlation coefficient R given higher weight, and proportion between asbestiform and non-asbestiform elongate particles is corrected by introducing powers ¼ and ½ for asbestiform and non-asbestiform particles respectively, making the required difference between the two groups more impactful for the fitting coefficient.

We also conditionally assume that in Eq. (6), parameter B= 1000. (Any other value can be used, considering the other two proportional coefficients A and C). Also, coefficient K was assumed to fluctuate between 1 and 5, coefficient T between 0.5 and 5, coefficient C between 1 and 10, and coefficient A between 0.10 and 2.

It should be noted that rigidity of elongate particles is another important determinant of their biological behavior. In this paper, we assumed that rigidity of serpentine, amphibole, and zeolite fibers listed in Table 4 are similar and the difference in biodurability of fibers can be measured by their relative biosolubility. It should be noticed, however, that rigidity is a function of fiber dimensions and elasticity and can differ between various particles.

### 2.8 Mahalanobis distance

We used Mahalanobis distance to evaluate the proximity of various datasets from the “asbestiform” and “non-asbestiform” combined sets, as a method to characterize the habit for each location/source of amphibole fibers in our study.

Only particles with length> 5 μm, width> =0.05 μm, width< =3 μm, length/width> =3:1 were considered.

The following formula was utilized:

(9) D = sqrt((μX - μY)^T * S^(-1)* (μX - μY)),

where X, Y are the two-dimensional vectors (length, width) respectively, of a dataset (X) and combined set of asbestiform (or non-asbestiform) particles (Y), ^T indicates matrix transposing, and S^(-1) is a pooled covariance matrix.

Mahalanobis distance is a metric of dissimilarities between datasets with multiple dimensions. We used this metric to compare asbestiform and non-asbestiform datasets. Then, each of the separate datasets in the study was compared with “total asbestiform” and “total non-asbestiform” data, to find the closely resembling “cloud” of particles. Mahalanobis distance provides additional confirmation of the proposed habit classification, proving objective character of the differences between dimensional characteristics of particles (sets) with different habits.

### 2.9 Prediction of mesothelioma potency

Mesothelioma potency factors are predicted in this study by using two major approaches.

First, potency will be modeled from the EMPA parameter for the dataset, as was outlined in Wylie et al. (2000). In particular, a linear model will be used from this publication as a regression equation:

(10) R_M_= -0.012+ 0.011EMPA

(R=0.988, R^2^=0.993, P < 0.01148).

Alternatively, dimensional coefficient of carcinogenicity (DCC) will be used, as in Eq. (7).

### 2.10 Estimation of cytotoxicity for elongate mineral particles

Estimations of cytotoxicity for elongate mineral particles were derived from published literature based on the membranolytic potential as measured by the Nolan method [32].

### 2.11 Estimation of mesothelial carcinogenicity in vivo

Results of the study were also compared to the outcomes of the intrapleural instillation study in rats for various mineral samples as reported by Cook et al. [33].

### 2.12 Other statistical considerations

The Support Vector Model was used for determination of decision boundaries in the classification process, with linear kernel approach and coefficient C= 1000.

Statistica 14.0 software was used for statistical calculations and visualization, Wolfram Alpha for calculus operations, and Crystal Ball^(T)^ for Monte Carlo simulations.

## 3 Results

### 3.1 Dimensional distribution of asbestiform and non-asbestiform tremolite EMPs

**Fig. 2:**
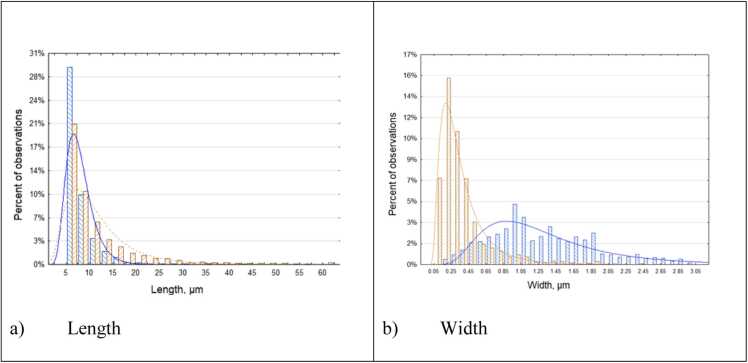
Dimensional distribution of length (a) and width (b) of EMPs from the testing sets. Blue – non-asbestiform and brown – asbestiform. Curves show fitted log-normal distributions.

Fig. 2 demonstrates the distribution of length and width of EMPs for asbestiform and non-asbestiform varieties of amphiboles in our study.

### 3.2 Major dimensional characteristics of testing, validation, and unclassified datasets

Major characteristics of particle populations in our study are shown in Table 5.

**Table 5:** Major characteristics of datasets in the study.

			Length, μm		Width, μm		Aspect ratio		Aerodynamic diameter, μm		EMPA, %		Surface area, μm2	
Dataset	Habit	Mineral type	Mean	Standard deviation	Mean	Standard deviation	Mean	Standard deviation	Mean	Standard deviation	Mean	Standard deviation	Mean	Standard deviation
Testing sets														
Udaipur, India	Asbestiform	Tremolite	9.30	1.18	0.77	0.09	30.53	4.26	3.67	0.40	0.027	0.033	18.15	5.03
Lone Pine, CA	Asbestiform	Tremolite	11.60	0.25	0.34	0.02	43.48	0.89	1.74	0.08	0.088	0.007	12.02	1.05
Yamaga Mine, Japan	Asbestiform	Tremolite	13.88	0.50	0.51	0.04	40.84	1.79	2.56	0.17	0.081	0.014	21.27	2.12
Jamestown, CA	Asbestiform	Tremolite	8.70	0.49	0.70	0.04	24.08	1.76	3.30	0.16	0.065	0.013	17.13	2.08
Korea	Asbestiform	Tremolite	11.40	0.51	0.35	0.04	48.31	1.84	1.77	0.17	0.147	0.014	11.93	2.18
HSE sample (1)	Asbestiform	Tremolite	9.74	0.49	0.30	0.04	46.31	1.78	1.54	0.17	0.269	0.014	9.25	2.10
HSE Sample (2)	Asbestiform	Tremolite	11.33	0.50	0.35	0.04	39.74	1.80	1.78	0.17	0.083	0.014	12.33	2.13
Metsovo, Greece	Asbestiform	Tremolite	8.65	0.91	0.32	0.07	52.84	3.29	1.56	0.31	0.306	0.025	7.62	3.89
Miners Bay, Canada	Non-asbestiform	Tremolite	8.42	2.17	0.84	0.17	10.59	7.80	4.08	0.73	0.000	0.060	28.09	9.23
Gouverneur, NY	Non-asbestiform	Tremolite	7.68	1.15	1.62	0.09	5.01	4.14	7.04	0.39	0.000	0.032	44.92	4.90
Sparta, NJ	Non-asbestiform	Tremolite	7.79	0.51	1.07	0.04	8.78	1.83	4.95	0.17	0.000	0.014	28.67	2.16
Madagascar	Non-asbestiform	Tremolite	8.06	0.25	1.31	0.02	7.06	0.90	5.83	0.08	0.000	0.007	36.80	1.07
Shinness, Scotland	Non-asbestiform	Tremolite	7.71	0.36	1.32	0.03	7.23	1.31	5.87	0.12	0.000	0.010	36.52	1.55
Dornie	Non-asbestiform	Tremolite	7.39	0.53	1.56	0.04	5.31	1.89	6.71	0.18	0.000	0.015	40.43	2.24
Swansea	Non-asbestiform	Tremolite	10.16	0.50	1.15	0.04	11.73	1.79	5.29	0.17	0.000	0.014	38.09	2.11
NIOSH cleavage fragments	Non-asbestiform	Tremolite	9.00	0.47	1.40	0.04	8.75	1.70	6.28	0.16	0.004	0.013	44.85	2.01
Validation sets														
Eastern New York	Asbestiform	Tremolite	13.68	0.75	0.23	0.06	120.56	2.71	1.15	0.25	0.648	0.021	10.87	3.21
Falls Village, CT	Non-asbestiform	Tremolite	7.55	1.69	1.10	0.13	10.30	6.10	4.95	0.57	0.000	0.047	30.53	7.22
Quebec	Non-asbestiform	Tremolite	7.47	2.27	0.96	0.18	8.36	8.19	4.49	0.76	0.000	0.063	23.14	9.68
Unclassified sets														
El Dorado Hills, CA	Unclassified	Tremolite	9.38	0.19	1.44	0.01	8.02	0.67	6.48	0.06	0.000	0.005	38.85	0.79
Ala di Stura, Italy	Unclassified	Tremolite	9.24	0.52	1.22	0.04	10.24	1.87	5.51	0.17	0.000	0.014	31.33	2.21
Brazil	Unclassified	Tremolite	7.45	0.29	1.31	0.02	7.31	1.04	5.77	0.10	0.000	0.008	33.81	1.23
Barstow, CA	Unclassified	Tremolite	11.64	0.18	0.69	0.01	32.26	0.65	3.28	0.06	0.080	0.005	25.08	0.77
Libby, MT amphiboles	Unclassified	Libby amphiboles	10.72	0.30	0.70	0.01	22.55	1.28	3.43	0.04	0.028	0.004	23.08	0.86

Table 6 contains the same set of parameters for asbestiform and non-asbestiform varieties of amphibole particles in testing sets, along with T test results for the difference between the two varieties.

**Table 6:** Characteristics of testing sets.

	Asbestiform		Non-asbestiform		P for T criteria
	Mean	Standard deviation	Mean	Standard deviation	
Length, µm	11.25	9.00	8.209	4.95	< 0.001
Width, µm	0.39	0.35	1.31	0.61	< 0.001
Aspect ratio	44.9	45.8	7.76	7.53	< 0.001
Aerodynamic diameter, µm	1.97	1.59	5.83	2.49	< 0.001
EMPA	0.14	0.35	0.00047	0.021	< 0.001
Surface area, µm^2^	13.15	17.91	37.30	32.98	< 0.001

### 3.3 Determination of DCC coefficients and the relationship with mesothelioma potency factors

As noted in the Materials and methods section, DCC is an empirical coefficient that combines several relevant parameters for mesothelioma risk determination. In general terms, DCC is a probabilistic expression (changing from 0 to 1, because of the exponential function 1-exp(-x) in its formula) that increases with surface area and decreases with width. It makes the coefficient an expanded form of specific surface area (that also increases with surface area and decreases with width). With parameter C being unequal to zero, DCC also approaches 0 where width is very small, accounting for elimination of the smallest particles from the lungs. At very small width level (not observed in the case of elongate mineral particles), surface area plays the pivotal role in the carcinogenicity; at larger width level, the width apparently becomes the driver of toxicity.

We determined the coefficients for DCC by maximizing the parameter Coef (Eq. (8)), utilizing Monte Carlo simulation.

**Fig. 3:**
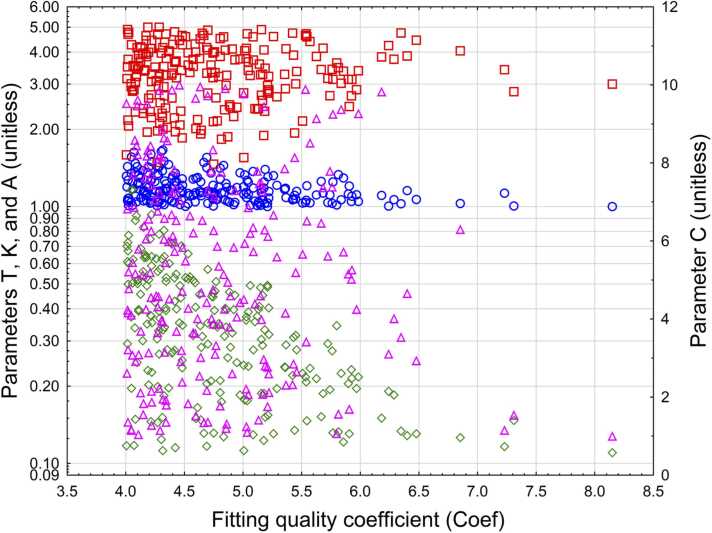
Relationship between fitting parameter Coef and various values of DCC parameters. Left axis: T, red squares; K, blue circles; A, green rhombuses. Right axis: C, magenta triangles.

The fitting process and results are illustrated in Fig. 3.

From here, we can determine the approximate values of the coefficients for the DCC, which provides at least a local maximum for the fitting coefficient:

T = 3, K= 1, B= 1000, C= 1, A= 0.11.

For this combination of parameters, we can develop the following regression equation:

(11) Log10(R_M_)= 0.04+ 1.15log10(Biosolubility)+ 2.15log10(DCC).

(R=0.98, R^2^=0.96, p < 0.006).

Table 7 shows the published and calculated values of mesothelioma potency R_M_ based on our model, along with the average DCC values for each mineral type.

**Table 7:** Published and calculated values of mesothelioma potency R_M_ (%).

Mineral type/Locations	Average DCC value	Mesothelioma potency, R_M_ (%), published	Mesothelioma potency, R_M_ (%), modelled
Chrysotile (Quebec)	0.085	0.0014	0.0013
Amosite (South Africa)	0.03	0.11	0.09
Crocidolite (Australia and South Africa)	0.11	0.52	1.18
Anthophyllite (Russia)	0.016	0.056	0.083
Libby amphiboles (Libby, MT)	0.021	0.03	0.025
Erionite (Karain, Turkey)	0.082	4.67	2.1

The ratio between average DCC values of asbestiform and non-asbestiform particles for the testing datasets in our study is 16.4.

### 3.4 Relationship between DCC and particle diameter, maximizing mesothelioma potency

It should be noted that for spherical particles, the expression

(12) A x Surface Area^K^ /(B x Width^T^ +C))

has a maximum by the width variable. It is easy to demonstrate that the value of Width maximizing DCC will be

(13) $$\mathrm{Width}={\frac{2\mathrm{KC}}{B(T-2K)}}^{1/T}$$

The parameters that we determine suggest the value of Width= 0.13 µm as the level, theoretically maximizing the DCC value. This estimation, though approximate, is in line with the conclusion from Korchevskiy and Wylie [25] and Wylie and Korchevskiy [4] demonstrating that Width= 0.15 μm is a cutpoint of width with the highest correlation with mesothelioma potency. With consideration of possible accuracy for width measurements (normally +/- 0.05 µm), this value most probably ranges from 0.1 to 0.2 μm. It means that fibers with maximum potency should have widths in the same range. The theoretical maximum value for Width in Eq. 13 confirms this observation.

### 3.5 Criteria fraction, Pearson Index, and DCC for amphibole datasets in our study

Table 8 shows criteria fraction, Pearson index, and average DCC value for all sets in our study.

**Table 8:** Criteria fraction, Pearson index, and average DCC value for all sets in the study.

Dataset	Habit	Mineral type	Criteria fraction	Pearson Index	DCC
*Testing sets*					
Udaipur, India	Asbestiform	Tremolite	0.459	0.29	0.027
Lone Pine, CA	Asbestiform	Tremolite	0.945	0.25	0.048
Yamaga Mine, Japan	Asbestiform	Tremolite	0.818	0.23	0.040
Jamestown, CA	Asbestiform	Tremolite	0.574	0.23	0.025
Korea	Asbestiform	Tremolite	0.914	0.13	0.057
HSE sample (1)	Asbestiform	Tremolite	0.934	0.21	0.058
HSE Sample (2)	Asbestiform	Tremolite	0.937	0.18	0.044
Metsovo, Greece	Asbestiform	Tremolite	0.887	0.28	0.068
Miners Bay, Canada	Non-asbestiform	Tremolite	0.273	0.54	0.005
Gouverneur, NY	Non-asbestiform	Tremolite	0.000	0.77	0.001
Sparta, NJ	Non-asbestiform	Tremolite	0.150	0.33	0.004
Madagascar	Non-asbestiform	Tremolite	0.082	0.67	0.002
Shinness, Scotland	Non-asbestiform	Tremolite	0.103	0.55	0.003
Dornie	Non-asbestiform	Tremolite	0.037	0.81	0.002
Swansea	Non-asbestiform	Tremolite	0.229	0.60	0.005
NIOSH cleavage fragments	Non-asbestiform	Tremolite	0.150	0.65	0.005
*Validation sets*					
Eastern New York	Asbestiform	Tremolite	0.945	0.17	0.138
Falls Village, CT	Non-asbestiform	Tremolite	0.278	0.55	0.007
Quebec, Canada	Non-asbestiform	Tremolite	0.100	0.39	0.003
*Unclassified sets*					
El Dorado Hills, CA	Unclassified	Tremolite	0.114	0.67	0.003
Ala di Stura, Italy	Unclassified	Tremolite	0.198	0.53	0.005
Brazil	Unclassified	Tremolite	0.125	0.35	0.003
Barstow, CA	Unclassified	Tremolite	0.648	0.35	0.033
Libby, MT amphiboles	Unclassified	Libby amphiboles	0.626	0.43	0.021

It can be demonstrated that DCC has a statistical relationship to criteria fraction and Pearson index parameters.

Specifically, for the testing sets, the following regression equation can be proposed:

(14) DCC= -0.004+ 0.06CriteriaFraction,

(R=0.96, R^2^=0.92, P < 0.000001),

and

(15) DCC= 0.06-0.087PearsonIndex

(R=0.82, R^2^=0.68, P < 0.00009).

It is noteworthy that tremolite sets have significantly different levels of DCC based on habit. The average DCC for an asbestiform testing set is 0.046 (standard deviation 0.015), and for a non-asbestiform is 0.0034 (standard deviation 0.0016). The T criteria value for two sets is 7.92, with P < 0.000002, indicating highly significant difference.

**Fig. 4:**
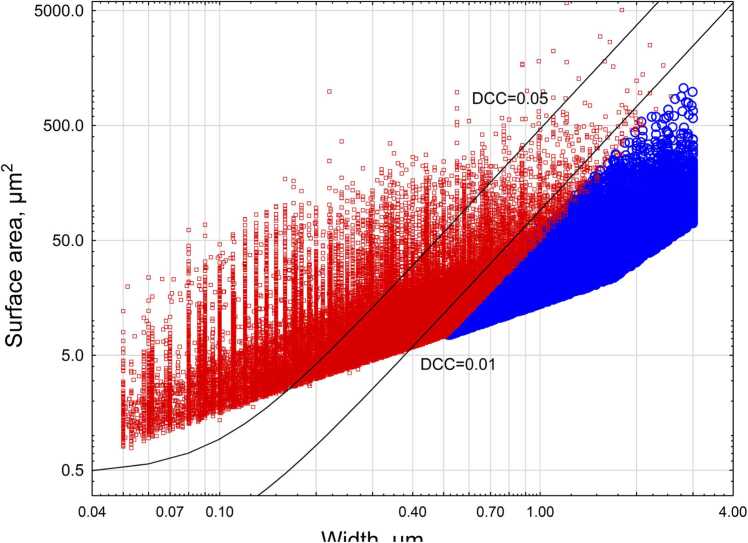
Relationship between width and surface area of criteria and non-criteria EMPs. Red squares – criteria particles, blue circles – non-criteria particles. Lines represent DCC metric at various levels: 0.05 and 0.01.

Fig. 4 shows relationship between DCC, width and surface area for criteria and non-criteria particles in the dimensional database, including EMPs with length> 5 µm, length/width> =3, width> =0.05 µm, width < =3 µm. Lines represent levels of DCC= 0.01 and DCC= 0.05. As we can see, all non-criteria particles have a DCC level lower than 0.01.

### 3.6 Prediction of amphibole habit based on dimensional parameters

We can demonstrate that the habit of amphibole datasets can be predicted based on dimensional parameters; specifically, fraction of criteria particles and Pearson Index.

**Fig. 5:**
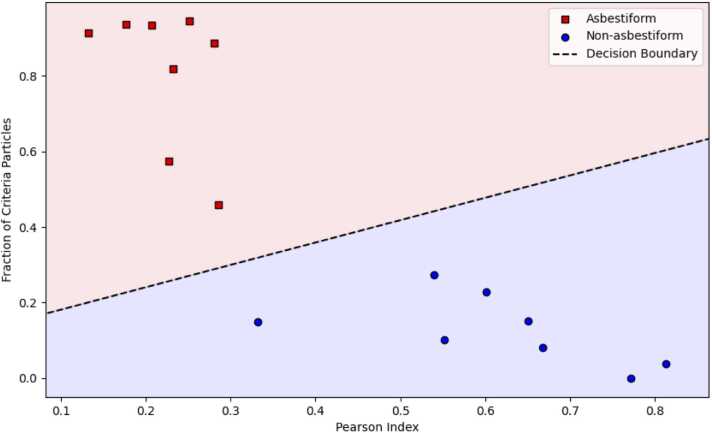
Discriminant analysis of asbestiform and non-asbestiform testing datasets.

We used vector discriminant analysis for the data from Table 8. Results of the discriminant analysis are demonstrated in Fig. 5.

The decision boundary is given as the following relationship:

(16) Criteria Fraction> =0.58PearsonIndex+ 0.12.

The error rate for the classification is zero. Table 9 provides the classification for testing, validation, and unclassified tremolite datasets.

**Table 9:** A-priori and a-posteriori classification of the tremolite datasets.

Dataset	Habit a-priori	Habit a-posteriori
*Testing sets*		
Udaipur, India	Asbestiform	Asbestiform
Lone Pine, CA	Asbestiform	Asbestiform
Yamaga Mine, Japan	Asbestiform	Asbestiform
Jamestown, CA	Asbestiform	Asbestiform
Korea	Asbestiform	Asbestiform
HSE sample (1)	Asbestiform	Asbestiform
HSE Sample (2)	Asbestiform	Asbestiform
Metsovo, Greece	Asbestiform	Asbestiform
Miners Bay, Canada	Non-asbestiform	Non-asbestiform
Gouverneur, NY	Non-asbestiform	Non-asbestiform
Sparta, NJ	Non-asbestiform	Non-asbestiform
Madagascar	Non-asbestiform	Non-asbestiform
Shinness, Scotland	Non-asbestiform	Non-asbestiform
Dornie	Non-asbestiform	Non-asbestiform
Swansea	Non-asbestiform	Non-asbestiform
NIOSH cleavage fragments	Non-asbestiform	Non-asbestiform
*Validation sets*		
Eastern New York	Asbestiform	Asbestiform
Falls Village, CT	Non-asbestiform	Non-asbestiform
Quebec, Canada	Non-asbestiform	Non-asbestiform
*Unclassified sets*		
El Dorado Hills, CA	Unclassified	Non-asbestiform
Ala di Stura, Italy	Unclassified	Non-asbestiform
Brazil	Unclassified	Non-asbestiform
Barstow, CA	Unclassified	Asbestiform
Libby, MT amphiboles	Unclassified	Asbestiform

As we can see, 100 % of asbestiform and 100 % of non-asbestiform testing sets were determined correctly by our method. The method also correctly identified 100 % of the sets in the validation group (Eastern New York sample as asbestiform, Falls Village, CT and Quebec tremolite as non-asbestiform).

The results for unclassified sets are also interesting. Barstow, California tremolite was indicated as asbestiform (fully confirming the conclusion from [7], along with Libby, Montana amphibole that is a confirmed mesothelial carcinogen.

At the same time, El Dorado Hills, California, Ala di Stura, Italy, and Brazil tremolites are determined to have characteristics of the non-asbestiform variety of tremolite. As was noted in the Materials and methods section, tremolite from Brazil was characterized as most probably byssolitic, or an intermediate habit of tremolite, that is not expected to have an asbestiform nature. Tremolite from Ala di Stura was characterized by Davis as a non-asbestiform variety, and it had to be “broken by hand to separate the thicker, longer bundles” (Davis et al., 1991). The habit of tremolite from El Dorado Hills, California is confirmed as non-asbestiform. As it will be demonstrated below, other methods can be applied for classification that also confirm this conclusion.

### 3.7 Mahalanobis distance between amphibole datasets and combined asbestiform and non-asbestiform groups parameters

Mahalanobis distance is a measure of proximity between a datapoint and a distribution, or between two distributions. We used length and width of individual particles as two dimensions. We calculated the distances between each of the datasets in the study with the combined distribution of length and width in asbestiform testing sets and non-asbestiform testing sets. We hypothesized that the distance with combined asbestiform distribution should be lower than with non-asbestiform distribution for asbestiform sets, and vice versa.

Results of the calculations are provided in Table 10.

**Table 10:** Mahalanobis distances between datasets and combined asbestiform and non-asbestiform length and width distributions.

Dataset	Habit	Mineral type	Distance from asbestiform distribution	Distance from non-asbestiform distribution	Predicted habit based on proximity to one of the groups
*Testing sets*					
Udaipur, India	Asbestiform	Tremolite	1.112	0.983	Non-asbestiform
Lone Pine, CA	Asbestiform	Tremolite	0.216	1.505	Asbestiform
Yamaga Mine, Japan	Asbestiform	Tremolite	0.414	1.646	Asbestiform
Jamestown, CA	Asbestiform	Tremolite	0.856	1.034	Asbestiform
Korea	Asbestiform	Tremolite	0.158	1.686	Asbestiform
HSE sample (1)	Asbestiform	Tremolite	0.324	1.651	Asbestiform
HSE Sample (2)	Asbestiform	Tremolite	0.160	1.683	Asbestiform
Metsovo, Greece	Asbestiform	Tremolite	0.360	1.668	Asbestiform
Miners Bay, Canada	Non-asbestiform	Tremolite	1.331	0.821	Non-asbestiform
Gouverneur, NY	Non-asbestiform	Tremolite	3.211	0.578	Non-asbestiform
Sparta, NJ	Non-asbestiform	Tremolite	1.706	0.393	Non-asbestiform
Madagascar	Non-asbestiform	Tremolite	1.583	0.035	Non-asbestiform
Shinness, Scotland	Non-asbestiform	Tremolite	1.779	0.119	Non-asbestiform
Dornie	Non-asbestiform	Tremolite	2.401	0.507	Non-asbestiform
Swansea	Non-asbestiform	Tremolite	1.757	0.477	Non-asbestiform
NIOSH cleavage fragments	Non-asbestiform	Tremolite	2.016	0.189	Non-asbestiform
*Validation sets*					
Eastern New York	Asbestiform	Tremolite	0.583	2.133	Asbestiform
Falls Village, CT	Non-asbestiform	Tremolite	2.058	0.351	Non-asbestiform
Quebec	Non-asbestiform	Tremolite	1.715	0.577	Non-asbestiform
*Unclassified sets*					
El Dorado Hills, CA	Unclassified	Tremolite	1.436	0.270	Non-asbestiform
Ala di Stura, Italy	Unclassified	Tremolite	1.886	0.276	Non-asbestiform
Brazil	Unclassified	Tremolite	1.642	0.175	Non-asbestiform
Barstow, CA	Unclassified	Tremolite	0.575	0.995	Asbestiform
Libby, MT amphiboles	Unclassified	Libby amphiboles	0.638	1.083	Asbestiform

**Fig. 6:**
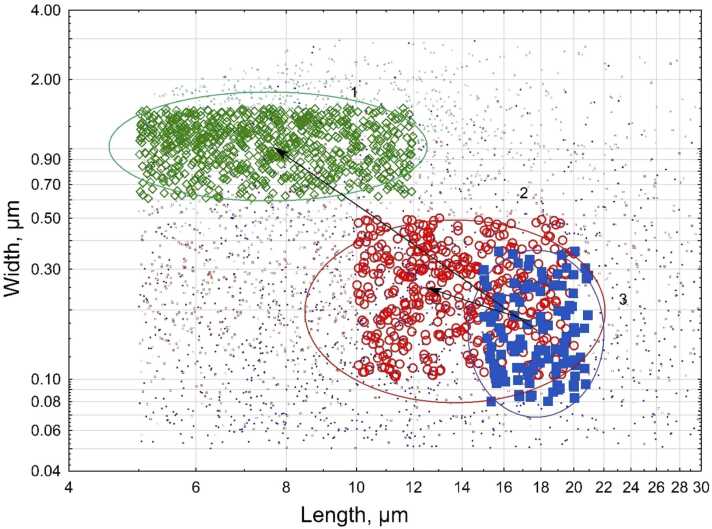
Illustration of Mahalanobis distance between various samples of elongate mineral particles (see text for explanation).

Fig. 6 conceptually demonstrates the use of Mahalanobis distance for samples of elongate mineral particles. The plot shows the “clouds” of elongate mineral particles, represented by their length and width. “Cloud 1” represents the most frequent length and width combinations for non-asbestiform EMPs, and “Cloud 2” for asbestiform EMPs. “Cloud 3” shows the validation sample of tremolite from Eastern New York. The arrows are the distances between Cloud 3 and Clouds 1 and 2. The distances can be measured by different metrics. Mahalanobis distance takes into account the variability of length and width for each of the samples.

The results of the Mahalanobis distance analysis are similar to what was determined by discriminant function modeling. From testing sets, there is only one tremolite location that was apparently misidentified: tremolite from Udiapur, India was found closer to non-asbestiform sets than to asbestiform. It yields a misclassification rate of 6.2 %. All validation sets are identified correctly. As previously, Barstow tremolite was indicated as asbestiform, and El Dorado Hills, Ala di Stura, and Brazil as non-asbestiform.

### 3.8 Determination of the habit for mixed samples

Our determination of the habit for amphiboles based on discriminant function (as well as the Mahalanobis distance approach) was made with the assumption that the samples are derived from a specific source, with the habit defined from a mineralogical standpoint with a certainty. In this case, prediction of potency for unclassified sets also assumes that we attempt to determine the typical habit for the sets, also with the assumption of the sample alignment and homogeneity. However, it is reasonable to assume that in many situations the analysis can be expanded to mixed datasets, or samples. The term “mixed” in this context refers to the situation when exposure is produced by particles from several sources, with varying habits.

We modelled mixing of the samples by deriving random samples from asbestiform and non-asbestiform testing sets for tremolite and determining criteria fraction, Pearson Index, and predicted habit based on the discriminant function method. We assigned the “asbestiform” category to the simulated sets when the fraction of particles from the asbestiform source was at least 1 % and “non-asbestiform” otherwise. We then combined the simulation results with the data for testing and validation sets in our study.

**Fig. 7:**
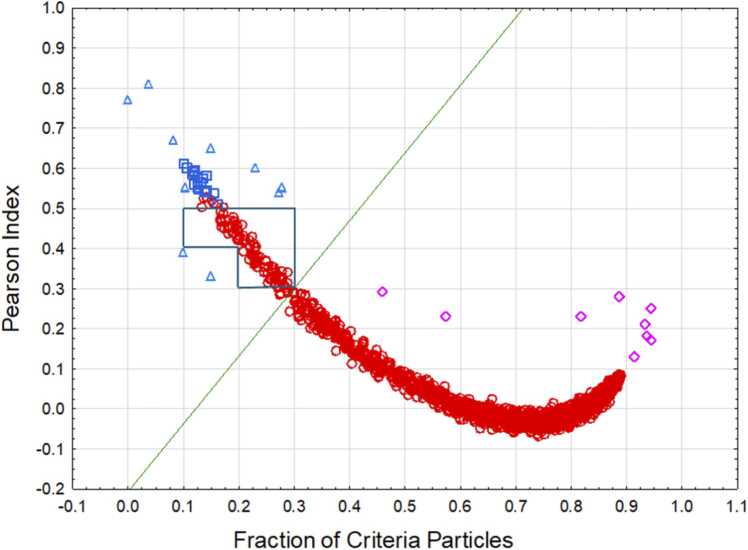
Results of the simulation study. Red circles – asbestiform simulated sets, blue squares – non-asbestiform simulated sets, magenta rhombuses – asbestiform testing and validation sets, light blue triangles – non-asbestiform testing and validation sets. The black line frame shows the area where the decision boundary method generates possible under-identification of asbestiform fraction.

Results of the simulation, along with the decision boundary provided by the discriminant function method, are demonstrated in Fig. 7.

We determined the area framed by a black line on Fig. 7 that should be additionally considered as “undetermined” category, even if the predicted fraction of asbestiform fibers in the samples would be very low. In this case, additional efforts should be taken to reevaluate the samples and determine their characteristics and homogeneity.

Combined decision rules for habit will be as follows:

If CriteriaFraction> =0.58PearsonIndex+ 0.12, **asbestiform**;

otherwise, if PearsonIndex> =0.3, PearsonIndex< =0.5, and CriteriaFraction> =0.2 and CriteriaFraction< =0.3,

or if PearsonIndex> =0.4, PearsonIndex< =0.5, and CriteriaFraction> =0.1 and CriteriaFraction< =0.2, **undetermined**,

otherwise, **non-asbestiform**.

The updated decision rules do not change the classification for testing, validation, or unclassified sets, as was previously proposed by the main decision boundary (see Table 9). In our study, about 4.3 % of randomly selected datasets would be identified as “undetermined”.

It is also possible to demonstrate that habit of datasets can be observed with a comparatively small selection of fibers. The error rate of the classification is not expected to exceed 1 % for mixed environments, if undetermined samples will be excluded and reanalyzed.

**Fig. 8:**
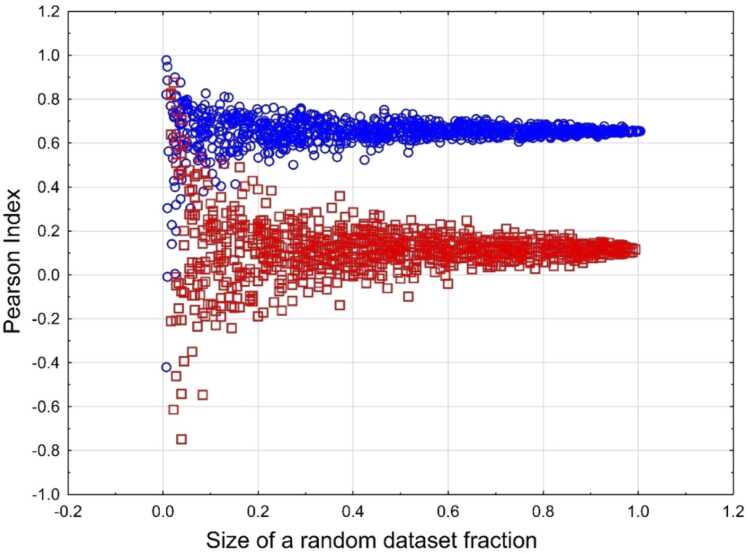
Distribution of Pearson Index for random selection of particle subsets from asbestiform and non-asbestiform fiber populations. Blue circles – samples from a non-asbestiform population, red squares – samples from non-asbestiform populations.

Fig. 8 shows the estimations of Pearson index for random samples derived from asbestiform (Korea) and non-asbestiform (NIOSH cleavage fragments) tremolite datasets.

As it can be seen, clear distinctions between two habits can be made at the fraction of about 10 % from the total dataset size.

### 3.9 Estimation of mesothelioma potency for various datasets

(10), (11) were used to estimate mesothelioma potency for tremolite sets in our study. Results are provided in Table 11.

**Table 11:** Modelled mesothelioma potency (R_M_, %).

Dataset	Habit	Mineral type	EMPA-based	DCC-based
*Testing sets*				
Udaipur, India	Asbestiform	Tremolite	0.018	0.039
Lone Pine, CA	Asbestiform	Tremolite	0.085	0.144
Yamaga Mine, Japan	Asbestiform	Tremolite	0.077	0.097
Jamestown, CA	Asbestiform	Tremolite	0.059	0.034
Korea	Asbestiform	Tremolite	0.150	0.203
HSE sample (1)	Asbestiform	Tremolite	0.284	0.209
HSE Sample (2)	Asbestiform	Tremolite	0.079	0.116
Metsovo, Greece	Asbestiform	Tremolite	0.325	0.296
Miners Bay, Canada	Non-asbestiform	Tremolite	−0.012	0.001
Gouverneur, NY	Non-asbestiform	Tremolite	−0.012	0.000
Sparta, NJ	Non-asbestiform	Tremolite	−0.012	0.001
Madagascar	Non-asbestiform	Tremolite	−0.012	0.000
Shinness, Scotland	Non-asbestiform	Tremolite	−0.012	0.000
Dornie	Non-asbestiform	Tremolite	−0.012	0.000
Swansea	Non-asbestiform	Tremolite	−0.012	0.001
NIOSH cleavage fragments	Non-asbestiform	Tremolite	−0.007	0.001
*Validation sets*				
Eastern New York	Asbestiform	Tremolite	0.701	1.362
Falls Village, CT	Non-asbestiform	Tremolite	−0.012	0.002
Quebec, Canada	Non-asbestiform	Tremolite	−0.012	0.000
Unclassified sets				
El Dorado Hills, CA	Unclassified	Tremolite	−0.012	0.000
Ala di Stura, Italy	Unclassified	Tremolite	−0.012	0.001
Brazil	Unclassified	Tremolite	−0.012	0.000
Barstow CA	Unclassified	Tremolite	0.075	0.065
Libby, MT amphiboles	Unclassified	Libby amphiboles	0.018	0.019

As we can see, the EMPA-based linear model predicts potency for non-asbestiform tremolite datasets as negative (negligible), vs. R_M_ for asbestiform variety, with an average of 0.134 % (slightly higher than for amosite, as estimated by [28]. The DCC-based model assigns low potency to non-asbestiform datasets (an average of 0.001 %, lower than for chrysotile). Both models point out to non-asbestiform varieties of tremolite as having non-significant potency for mesothelioma. The highest potency (higher than for crocidolite) is estimated for the tremolite variety from Eastern New York.

### 3.10 In-vitro toxicological characteristics of amphiboles

Nolan et al. [8] reported results of in vitro tests for red blood cell cytotoxicity induced by various types and habits of mineral fibers. Nolan’s study reported HC50 as a value in mg/ml concentrations damaging 50 % of the cells such that a higher level of HC50 corresponds to a lower level of cytotoxicity. Nolan et al. suggested that the ability of mineral specimens to alter the permeability of a population of human erythrocytes is one of the toxicity metrics for fibers. It should be noted, however, that hemolytic potential is not a direct determinant of fibrogenicity of mineral dusts [34]. Also, Blum et al. [35] and Henzi et al. [36] promoted the idea that factors protecting mesothelial cells from asbestos cytotoxicity (like overexpression of calretinin) may contribute to mesothelioma development.

We recalculated Nolan’s HC50 values in mg/ml to corresponding values of concentration in fibers x10^9^ per ml for various samples of tremolite. We included Libby amphibole in the analysis as one of the analogies of tremolite with extensive toxicological data available.

The HC50 values are shown in Table 12.

**Table 12:** HC50 of fibers.

Mineral type, location.	HC50, mg/ml	HC50, fibers x10^9^/ml
Tremolite, Udaipur, India	2.81	0.41
Tremolite, Jamestown, CA	1.14	0.17
Tremolite, Korea	1.38	0.51
Tremolite, Metsovo, Greece	3.06	1.7
Tremolite, Swansea	2.53	0.09
Libby, MT amphiboles	2.07	0.22

**Fig. 9:**
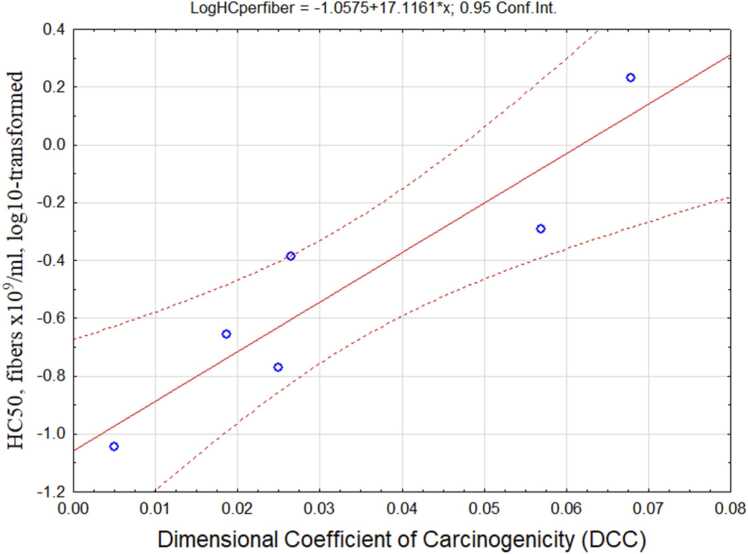
Relationship between DCC and cytotoxicity of elongate particles.

We determined that log10-transformed HC50 per fiber count correlates with the DCC parameter as demonstrated in Fig. 9.

The following regression equation was derived:

Log10(HC50)= -1.06+ 17.12DCC

(17) (in previous notation) (R=0.927, R^2^=0.86, p < 0.00776).

As can be seen, HC50 increases with DCC, while cytotoxicity per fiber decreases, and mesothelioma potency increases. This relationship illustrates the idea that mesothelial carcinogenicity is competing with cytotoxicity, probably because cells with mutations are being removed by cytotoxic processes, induced by inflammation.

### 3.11 In-vivo toxicological characteristics of amphiboles

Our model and conclusion can also be tested on the data for the intrapleural instillation of various mineral fiber samples in Wistar rats as reported by [33]. We used relative potency estimations for several types of mineral fibers. We averaged the data for all subtypes of crocidolite. Table 13 contains relative mesothelioma potency factors in rats reported by Cook, for the dose of 20 mg. We used the depositary from the U.S. EPA to obtain dimensional characteristics of the samples in the Cook paper. We also list average DCC values, biosolubility values, and mesothelioma potency factors R_M_ calculated for each sample by Eq. (12) in this paper.

**Table 13:** The results of the modeling of in vivo potency for samples of different mineral fibers.

Mineral type	Relative potency by [33]	Average DCC	Biosolubility by Gualtieri	R_M_, %, modeled by Eq. (12)	Relative potency modelled by Eq. (17)
Crocidolite	1.22	0.089	66	0.748	1.20
Amosite	1.00	0.041	74	0.161	0.88
Tremolite	0.66	0.032	49	0.059	0.67
Anthophyllite	0.47	0.0139	245	0.062	0.68
Non-fibrous grunerite	0.1	0.0066	74	0.003	0.06

We initially excluded tremolite from the statistical calculations and determined that potency by Cook in rats highly correlated with log10-transformed potency in humans. The regression equation is:

(18) Relative potency in rats = 1.26+ 0.48log10(R_M_),

R= 0.959, R^2^= 0.92, p < 0.04.

For example, this equation for tremolite allows sufficient accuracy in the estimation of tremolite potency in rats: 0.67 vs. reported 0.66.

## 4 Discussion

Studies of amphibole minerals have significant importance for toxicological science and environmental protection. Amphibole exposure has been reported for many populations as demonstrated in fiber lung burden studies [37], [38].

However, amphiboles exist in different habits, some of them massive (or non-asbestiform) and some asbestiform. Presence of the third (byssolitic) habit has been also reported [12], [39], [7].

It is the result of long-term observations that allows us to hypothesize that the habit of fibrous minerals has dramatic toxicological implications. For example, for mesothelioma, non-asbestiform tremolite appears not to be a significant risk factor in the absence of the asbestiform admixture or contamination. Local mesothelioma epidemics such as found in Metsovo, Greece, provide other examples of observations in this regard. For example, several authors have addressed the issue of elevated mesothelioma incidence (reported as 300 times higher than the baseline) in the area of Metsovo, a complex of small villages in the prefecture of Ioannina in north-west Greece, which was found to be related to the tremolite content in locally used whitewash [40]. At the same time, tremolite from Trikala (Megarchi), Greece, close to the Metsovo area, but with presence of an apparently non-asbestiform (called “crystalline”) form of tremolite was reported as not associated with elevated mesothelioma (though apparently causing pleural plaques) [41].

There are numerous biological reasons for asbestiform and non-asbestiform particles to produce different toxicological effects, or levels of toxicological effects. Non-asbestiform particles are shorter, while length is one of the most important determinants of toxicity for elongate structures [42], [43]. The diameter of particles is dramatically different for asbestiform and non-asbestiform varieties, leading to differences in toxicokinetics, and also in specific signaling pathways when interacting with cells [4], [44]. Asbestiform particles may have “needle-like” geometry that was implicated in many models of toxicity [45]. Also, specific surface area of non-asbestiform particles is significantly lower than for the asbestiform variety that decreases biological activity and relevance for adverse cellular response.

However, knowledge of the toxicological properties of many types of amphiboles remains limited. No epidemiological cohort data are available for tremolite or actinolite, specifically. Tremolite as a contaminant of chrysotile was reported as one of the factors that increased the mesothelioma rate in Quebec chrysotile mines, with the risk level apparently different for various mining areas depending on the tremolite content [47], [46]. Tremolite in the Quebec chrysotile mines was suggested to have asbestiform habit [48], or at least a mixture of asbestiform and non-asbestiform variety.

The landmark work of Nolan et al. [8] significantly elevated the scientific knowledge on the difference between various habits of amphiboles (specifically, tremolite). However, this work used in vitro tests for determination of cytotoxicity rather than carcinogenicity of tremolite.

In this paper, we developed an approach to evaluate the habit of amphibole samples from the position of its toxicological implications.

As testing sets, we used 8 asbestiform and 8 non-asbestiform datasets of tremolite from different locations. The determination of habit was performed from mineralogical consideration based on mineralogical literature and geological assessment.

Specifically for this study, validation data were developed by laboratory analysis of one asbestiform and two non-asbestiform datasets. For these datasets, dimensional counts were performed, and mineralogical determination of habit was also made. Several datasets were included in the analysis as unclassified. Some of the unclassified datasets were suggested to have a byssolitic habit, and some had evidence of potential mixed habit. The data were combined and added to a database that was previously described in literature [5].

We determined statistical characteristics for the datasets in the study. In particular, we demonstrated that length and width of asbestiform and non-asbestiform datasets have distinct distributions (Fig. 2). It is especially revealing for width, where distribution curves only partially overlap.

We reported several dimensional characteristics for each dataset, including average length, width, aerodynamic diameter, aspect ratio, surface area, and EMPA, the fraction of elongate particles thinner than 0.15 µm from all particles longer than 5 µm. We demonstrated that all listed parameters show statistically significant differences between asbestiform and non-asbestiform sets. For example, average aerodynamic diameter, calculated by the Timbrell method, is 1.97 µm for asbestiform testing sets (standard deviation 1.59 µm) vs. 5.83 µm for non-asbestiform sets (standard deviation 2.49 µm). This puts the central tendency values for two habits of tremolite on two sides of the usual criteria for respirable dust (4 µm), confirming the significant difference between two groups, although the distributions can still overlap in respirable range.

We determined several major characteristics of amphibole datasets that provide in-depth evaluation for their dimensional distributions. In particular, the fraction of so-called criteria particles was determined. Wylie et al. [5] demonstrated that individual elongate mineral particles can be distinguished for their most probable habit, based on length and width; the criteria particles have an approximately equal error rate of about 15 % for both asbestiform and non-asbestiform sets. At the same time, as demonstrated by Van Orden [1], the error rate of this methodology varies for different mineral types. However, the fraction of criteria particles satisfying the discriminant equation from Wylie and Korchevskiy [4], independently from a mineral type, enables the distinction between populations of asbestiform and non-asbestiform habits.

Another characteristic of the datasets, indicative of their habit, is the Pearson index – the correlation coefficient between logged length and width of elongate particles. This parameter is significantly lower in asbestiform datasets than in non-asbestiform, which can be explained by their origin. Asbestiform fibers are grown in a natural geological environment, and the probability of their being of a specific width is statistically independent from the length. To the contrary, elongate particles from massive mineral sources are produced by mechanical forces that reduce both length and width, creating a correlation between these two dimensions.

In our study, we used vector analysis to develop predictive decision boundaries for Pearson index and fraction of criteria particles as universal metrics of the habit. The model allows for perfect discrimination of testing datasets in the study (0 % error rate). The method also perfectly determined the habit of three validation sets. It also allowed realistic estimations of the prevailing habit for the unclassified datasets. In particular, we determined that tremolite from Brazil was non-asbestiform, though fibrous, most probably displaying a byssolitic habit. The set of tremolite from Barstow was confirmed as having an asbestiform nature.

It should be noted that the development of the proposed classification methodology should not be considered “circular”, because initial classification of the testing and validation datasets as asbestiform or non-asbestiform was performed based on mineralogical and geological information rather than pure dimensional criteria. The major research question for this study was to determine if mineralogical classification can be efficiently replicated by a “blind” mathematical analysis, using only length and width of particles, individually or as a dataset. The study confirmed that a conclusion about the tremolite sample habit can be made just based on the dimensional data. It allowed to clarify the toxicological significance of the habit that appeared to be systematically related to measurable characteristics of particles, and not to some unspecific geological or mineralogical characterization.

Based on the success of the classification of tremolite datasets according to their dimensions, we addressed the issue of classification for mixed samples. We performed a simulation creating mixed datasets from asbestiform and non-asbestiform testing sets in our study. We developed an approach that allowed us to select samples with “undetermined” habit, if they have fraction of criteria particles and Pearson index within specific ranges. This category reflects the possibility that some mixed datasets of tremolite particles can have a mid-range Pearson index with a low fraction of criteria particles. Such sets would be characterized as non-asbestiform by decision boundaries, but they still might have up to 1 % of asbestiform component, requiring their separate classification and potentially additional analysis.

In practical situations, the model allows recognition of a combination of factors that may provide evidence of non-homogenous sources. Such examples may include airborne samples when asbestos fibers are present as an inclusion in massive minerals, and when comminution produces a combination of habits instead of one certain type of exposure. Further studies will be needed to replicate specific conditions that would be typical for “mixed” environments. The simulation performed in this study demonstrates that a health-protective approach can be proposed when a sample would be reanalyzed if quantitative parameters would not be fully consistent to fully substantiate non-asbestiform habit.

Alternatively, we tested another approach to the classification of amphibole samples. We utilized Mahalanobis distance as a metric of distance between a sample and distribution. We determined two populations: asbestiform and non-asbestiform particles. Each set of the study was explored from the position of the distance to the centroids of the populations. We demonstrated that this approach is also efficient in determining the dataset habits (though one of the asbestiform sets was misclassified by the Mahalanobis distance approach). All other classifications that confirm our conclusions about the major statistical differences between asbestiform and non-asbestiform particles were made correctly.

To evaluate mesothelial carcinogenicity of amphibole samples, we used two approaches. One of them was fully based on criteria EMPA that was shown to be highly predictive of mesothelioma potency for amphibole fibers [49]. The second criterion was based on the metric called dimensional coefficient of carcinogenicity (DCC), which is a function of surface area and width: two parameters that are interrelated, but reflect different aspects of the carcinogenicity of fibers. In particular, surface area was suggested to be a predictor of inflammogenic potential. For example, a good illustration of this fact can be found in Donaldson et al. [50] where the inflammation potential of “long” amosite fibers was higher than the one for the UICC amosite sample, which in its turn was higher than the potential for “short” amosite fibers; the values of corresponding median surface area of fibers can be estimated as respectively 13.6 µm^2^, 4.95 µm^2^, and 2.89 µm^2^. The Pearson correlation between surface area and inflammatory markers at the second day after peritoneal instillation of fibers in Donaldson’s experiment was 0.92 (P = 0.06). The Spearman rank correlation was 1.0.

To the contrary, many factors of carcinogenicity were demonstrated to be inversely proportional to width of fibers, or proportional to corrected aspect ratio, that is inversely proportional to width, with length being constant [26], [4].

DCC is a probabilistic expression that increases with surface area and decreases with width. It makes the coefficient a mathematical transformation of the specific surface area (that also increases with surface area, and decreases with width). With parameter C being unequal to zero, DCC also approaches 0 for very small widths. At very small widths, surface area plays the pivotal role in the carcinogenicity; at larger widths, width appears to be the driver of toxicity.

It can be also demonstrated that DCC is an efficient discriminant factor for carbon nanotubes (CNTs) allowing distinction between CNTs with reported mesotheliomagenic potential, vs. non-mesotheliomagenic CNT (Korchevskiy and Wylie, unpublished).

DCC includes several coefficients, which we estimated based on various considerations. We assumed that DCC with the correct parameter should (a) yield the highest correlation with mesothelioma potency for chrysotile, crocidolite, erionite, amosite, anthophyllite, and Libby amphiboles, with relative biosolubility of fibers used as a second independent variable, and (b) provide efficient differentiation between asbestiform and non-asbestiform particles. We developed a coefficient for fitting maximization, including correlation with potency and average DCC value for asbestiform and non-asbestiform particles in the testing set for our study. Using Monte Carlo simulation, we determined at least a local maximum for the fitting coefficient, providing a high level of correlation with potency (R=0.98, p < 0.006), and the high ratio between average DCC values for asbestiform and non-asbestiform particles. Average DCC is 0.046 for asbestiform sets, and 0.003 for non-asbestiform sets. It should be noted that DCC has a probabilistic nature because of the exponential function in the formula, making DCC fluctuate between 0 and 1 depending on surface area and width of particles. It can be supposed that DCC is a surrogate for a “mesothelioma probability”, though this understanding is quite conditional. Still the DCC = 0.05 or 0.01 can be seen as convenient indicators of asbestiform vs. non-asbestiform (or mesotheliomagenic vs. non-mesotheliomagenic) sets.

As a result, we were able to make two estimates of potency, based on a linear (with EMPA) and a non-linear (with DCC) method. The correlation between two estimations is 0.95 (R^2^=0.90, p < 0.0001). The EMPA method determined positive mesothelioma potency for asbestiform sets, and negative (negligible) for non-asbestiform sets. The DCC method assigned some low levels of potency to non-asbestiform elongate particles, but it is lower than for chrysotile, also evidence of a negligible or non-significant level. The average values of the two estimates of potency also yield negative (negligible) potency for non-asbestiform particles.

We also determined that DCC parameters are inversely related to the metric of fiber cytotoxicity, measured in terms of Nolan’s HC50 parameter, expressed as number of fibers in the exposure per ml of erythrocytes suspension producing 50 % membranolytic potential. Obviously, there is no direct relationship between mesothelioma development and cytotoxicity in the red blood cells. We confirmed by modeling that mesothelioma potency for various types of mineral fiber is inversely related to the cytotoxic potential measured by Nolan. The negative correlation between this cytotoxicity metric and mesothelioma potency was demonstrated earlier [51]. This finding is in agreement with our general hypothesis that mesothelial potency of amphibole fibers is driven by two concurrent processes: inflammation and cytotoxicity, vs. possible immune suppression or “immortalization” of cells with mutations, caused most probably by penetration of fibers to the nucleus of macrophages (and/or mesothelial cells) [52].

We also confirmed the results of our study with mesothelioma potency in Wistar rats after intrapleural installition of various types of mineral fibers. We demonstrated that the relative potency for in vivo tests can be reconstructed by the R_M_ values as modeled according to the methodology proposed in this paper. In particular, for tremolite (the mineral type used for testing and validation sets in our study), good accuracy of prediction was achieved (relative potency of 0.67 by our method vs. 0.66 reported by [33].

While tremolite datasets were selected for the testing and validation sets in our study, the results have direct implications for other types of amphiboles. For example, as it was shown in this paper, mesothelioma potency modeling based on EMPA and DCC worked efficiently for other amphiboles (crocidolite, amosite, anthophyllite), but also for serpentines (chrysotile) and zeolites (erionite).

It should be noted that the results of this study relate to specific counting criteria of elongate particles. As a rule, we utilized the count of particles with length> =5 µm, length/width> =3:1, width> =0.05 µm, width< =3 µm for our analysis. For determination of Pearson index, we used a wider range of length, limiting it to length> =2 µm. Inclusion of only particles with aspect ratio greater or equal to 5:1, as it is done in some standards, significantly limits the power of statistical analysis, and may artificially bias the data toward the asbestiform variety of elongate particles. Additional research would be needed to determine the methods of correction for dimensional data with higher aspect ratio for the models in this paper to be utilized.

Our preliminary estimates show that the formula

(19) Criteria Fraction* >= 5.8PearsonIndex* -2.02

where CriteriaFraction* is a fraction of criteria particles among elongate particles with aspect ratio of greater or equal to 5:1,

PearsonIndex* is a Pearson Index for this category of particles,

provides an acceptable error rate (about 6.3 %) for determination of asbestiform datasets.

It was also determined that DCC parameter correlates with Pearson index and criteria particles fraction for amphibole datasets. From here we can conclude that habit and toxicological potential of fibers are deeply interrelated. These two processes (habit-forming and carcinogenicity) are dependent on the same parameters: longer, thinner shape is what makes EMPs carcinogenic. The same process determines the classification of amphiboles by habit. This allows us to combine the analysis of mineralogical and toxicological processes and to predict the behavior of amphibole samples in toxicological experiments and in epidemiological observations based on the origin and geological history of specific deposits.

Our study has uncertainties and limitations. While the authors believe that the selection of testing datasets for our modeling was adequate and representative, more samples can be included for further analysis to check for consistency of the results and conclusions. Tremolite was selected as a valid example of the amphibole widely represented by asbestiform and non-asbestiform varieties. However, other types of minerals may have different qualities and characteristics that would be important to study. The decision boundary model as evaluated in this paper is recommended for tremolite at this point, though there is strong evidence that its average efficiency would be high for other types of amphiboles and single-chain silicates as well (see [5]. Our preliminary results for other mineral types demonstrate an error rate at 0 % for crocidolite vs. riebeckite, and amosite vs. grunerite. The error rate is about 5 % for other minerals.

The biases of our approach can be seen in the limited volume of epidemiological studies available. However, we tested the developed model for various sources of data (human epidemiology, in vitro studies, in vivo experiments), and see sufficient consistency. The bias of proposed dimensional metrics can be seen in their empirical, rather than fully theoretical basis. In particular, the main challenge of the DCC metric is in determination of its coefficients. The authors are confident, nevertheless, that DCC reflects an objective mode of action for elongate mineral particles in mesothelioma development.

Mathematical modeling used in this paper also has its limitations in statistical power. However, practical application of our models for validation and additional, non-classified, sets demonstrated reasonable agreement with mineralogical and toxicological considerations. The limitations of our approach should be taken into account when they would be applied to risk assessment in humans. However, it seems that relationships between toxicological effects and dimensional characteristics of elongate particles, discovered in our analysis, are based on the deep biological processes in the human body that is expected to be confirmed in future observations and experiments.

Further studies are needed to develop the proposed methodology of quantitative analysis and toxicological evaluation of elongate mineral particles. Potential testing of the methodology on an expanded set of samples, including other mineral types of amphiboles, would be beneficial. It will be important to test the DCC metric as a correlating parameter for in vivo studies of mesothelioma in animals. It also should be expected that a public health protection policy should better accommodate the most recent toxicological studies demonstrating that elongate mineral particles have distinct toxicological properties depending on the size and morphology. Differentiation between truly dangerous types of asbestiform amphiboles and non-asbestiform analogies, resembling nuisance dust, should be helpful for correct prioritization and focused protection of workers and populations from carcinogenic exposure.

## Author statement

All authors have seen and approved the final version of the manuscript being submitted. The article is the authors’ original work, hasn’t received prior publication, and is not under consideration for publication elsewhere.

## CRediT authorship contribution statement

**Ann G. Wylie:** Writing – review & editing, Writing – original draft, Validation, Methodology, Conceptualization. **Andrey A. Korchevskiy:** Writing – review & editing, Writing – original draft, Validation, Supervision, Software, Methodology, Formal analysis, Conceptualization.

## Declaration of Competing Interest

The authors declare the following financial interests/personal relationships which may be considered as potential competing interests: The paper used the results of modeling and other analysis as part of the research project on toxicological characterization and human health risk assessment for potential associated minerals in the cosmetic grade talc deposits, supported by J&J. No time for the writing of this paper was billed to any clients of the authors. No copies of the paper were provided to any clients of the authors prior to the publication. No feedback or comments were received from any party outside of the authorship team during the paper preparation. Authors disclose that they have participated in the litigation of matters related to asbestos and/or talc as consultants or experts.

## Data Availability

Data will be made available on request.

## Glossary

Criteria particles: Elongate mineral particles with length and width that would satisfy the Eq. 2.99log10(Length)-5.82log10(Width)-3.80 > =0
DCC (Dimensional Coefficient of Carcinogenicity): A probabilistic metric of dimensions for elongate mineral particles, quantified as 1-exp(-0.11 Surface Area /(1000width^3^ +1))
EMPA: The fraction of elongate mineral particles with width equal or less than 0.15 μm among all elongate mineral particles longer than 5 μm
HC50: The concentration of particles required to lyse 50 % of the erythrocytes in a suspension containing 1.8 × 10^8^ cells/ml
Mahalanobis distance: Mathematical measure for the proximity of multi-dimensional datasets
Mesothelioma potency: A quantitative metric of the rate of mesothelioma mortality per unit of cumulative exposure. In this paper, refers to R_M_ (%) value as calculated by Hodgson, Darnton (2000)
Pearson index: The linear correlation coefficient between log-transformed length and width of particles.
